# Extensive Transcript Diversity and Novel Upstream Open Reading Frame Regulation in Yeast

**DOI:** 10.1534/g3.112.003640

**Published:** 2013-02-01

**Authors:** Karl Waern, Michael Snyder

**Affiliations:** * Department of Genetics, Stanford University School of Medicine, Stanford, California 94305; † Department of Molecular, Cellular, and Developmental Biology, Yale University, New Haven, Connecticut 06520

**Keywords:** yeast, RNA-sequencing, environmental conditions, UTRs

## Abstract

To understand the diversity of transcripts in yeast (*Saccharomyces cerevisiae*) we analyzed the transcriptional landscapes for cells grown under 18 different environmental conditions. Each sample was analyzed using RNA-sequencing, and a total of 670,446,084 uniquely mapped reads and 377,263 poly-adenylated end tags were produced. Consistent with previous studies, we find that the majority of yeast genes are expressed under one or more different conditions. By directly comparing the 5′ and 3′ ends of the transcribed regions, we find extensive differences in transcript ends across many conditions, especially those of stationary phase, growth in grape juice, and salt stimulation, suggesting differential choice of transcription start and stop sites is pervasive in yeast. Relative to the exponential growth condition (*i.e.*, YPAD), transcripts differing at the 5′ ends and 3′ ends are predicted to differ in their annotated start codon in 21 genes and their annotated stop codon in 63 genes. Many (431) upstream open reading frames (uORFs) are found in alternate 5′ ends and are significantly enriched in transcripts produced during the salt response. Mutational analysis of five genes with uORFs revealed that two sets of uORFs increase the expression of a reporter construct, indicating a role in activation which had not been reported previously, whereas two other uORFs decreased expression. In addition, RNA binding protein motifs are statistically enriched for alternate ends under many conditions. Overall, these results demonstrate enormous diversity of transcript ends, and that this heterogeneity is regulated under different environmental conditions. Moreover, transcript end diversity has important biological implications for the regulation of gene expression. In addition, our data also serve as a valuable resource for the scientific community.

Regulation of gene expression occurs on many levels, both transcriptionally and posttranscriptionally. Measuring RNA levels as a proxy for protein levels and protein function is inadequate. In yeast, RNA levels and protein levels do not perfectly correlate, instead having an *r^2^* value of 0.77 (Lee *et al.* 2011). Thereby, there must be features of messenger RNAs (mRNAs) or the RNA-handling machinery that affects mRNA levels, translational efficiency, and/or protein turnover.

It has been known for some time that untranslated regions (UTRs) play a significant role in the regulation of gene expression (*e.g.*, van der Velden and Thomas 1999). Sequences in UTRs may control translation, subcellular localization, and transcript stability through a variety of mechanisms (*e.g.*, Mignone *et al.* 2002). The UTR of a transcript often changes through the use of differential transcription start sites [*e.g.*, (Law *et al.* 2005). These differential transcript ends—referred to as differential ends in this article—may thus have a role in posttranscriptional regulation of gene expression.

The yeast *Saccharomyces cerevisiae* provides an ideal organism in which to study this phenomenon. A wealth of molecular, genetic, and genomic information exists for *S. cerevisiae*, much of it assembled and conveniently available at the Saccharomyces Genome Database (Cherry *et al.* 1998). Although the complete genome sequence has been known since 1996 (Goffeau *et al.* 1996), information on the exact length of mRNAs has historically been scarce. Several investigators have attempted to provide this information by using cDNA sequencing (Miura *et al.* 2006), microarrays (David *et al.* 2006), Illumina-based RNA sequencing (Nagalakshmi *et al.* 2008), Illumina-based 3′ RNA-Seq (Yoon 2010), and Helicos-based RNA sequencing for 3′ ends (Ozsolak *et al.* 2010). Furthermore, previous studies in which authors examined alternative UTRs in yeast have only considered cells growing in one condition, with the exception of the studies by Miura *et al.* and Yoon *et al.*, whereby analysis was performed under two or three different conditions, respectively (Miura *et al.* 2006; Yoon 2010). Miura *et al.* found that most genes had multiple 5′ and 3′ ends, but they did not examine how these were regulated under different incubation conditions in the same strain. A large-scale systematic analysis of differential transcript ends under varying environmental conditions and stressors has thus not previously been performed. Our study examined not only which and how many transcripts have alternate forms but also how differential ends vary under a large set of different conditions and how these differential ends affect gene expression.

There has long been evidence that alternative 5′ UTR sequences in mRNAs can have biological relevance by affecting protein synthesis or protein function. Previous authors have observed that transcripts in yeast may vary in length and result in (1) a relative increase in protein level (Law *et al.* 2005), (2) a decrease in protein level (Law *et al.* 2005), (3) secretion of a particular protein (Carlson and Botstein 1982), and (4) an entirely different protein from an overlapping out-of-frame open reading fame (ORF) (Guittaut *et al.* 2001; Coelho *et al.* 2002; Kumar *et al.* 2002). Existing knowledge on alternative UTRs is compiled in several reviews, such as (van der Velden and Thomas 1999; Mignone *et al.* 2002; Landry *et al.* 2003; Kochetov 2006).

RNA sequencing (RNA-Seq) provides a powerful tool to study alternative UTRs. Previous studies (Nagalakshmi *et al.* 2008; Yoon 2010; Ozsolak *et al.* 2010) have used RNA-Seq to study the yeast transcriptome in a few conditions and identified many genes with multiple 3′ transcript boundaries. RNA-Seq provides much better resolution than microarrays (Wang *et al.* 2009), and differential transcript ends have been identified in RNA-Seq data by examining expression drop-offs (Nagalakshmi *et al.* 2008); in addition, poly-adenylation in eukaryotic transcripts can be identified in RNA-Seq data as “end tags,” runs of adenines that do not map back to the genome that lie as the ends of “mappable sequences” (see *e.g.*, Wang *et al.* 2009) for an explanation).

This study generated deep RNA-Seq data from yeast incubated under many different environmental conditions and stresses, selected to activate a broad variety of gene expression patterns and regulatory networks. We focused on differential transcript ends and found that alternative UTRs are pervasive in yeast. Importantly, we also found that the uORFs introduced in at least two genes with longer 5′ ends enhance gene expression.

## Materials and Methods

The BY4741 strain used in (Nagalakshmi *et al.* 2008) was used in this study. RNA-Seq was performed using the protocol developed in (Nagalakshmi *et al.* 2008), further described in (Nagalakshmi *et al.* 2010) and (Waern *et al.* 2011), and using the modifications developed by (Parkhomchuk *et al.* 2009) to generate strand-specific reads.

Analysis was performed on custom software developed in-house using BowTie (Langmead *et al.* 2009) to map reads to the S288C reference genome available on SGD, downloaded on May 17, 2010. Python, NumPy, SciPy, and matplotlib were used to further process the data. The software’s source code is available (*Saccharomyces Genome* Database). Of note, to maximize the information gleaned, unmappable reads were trimmed by four bases from the 3′ end and remapping was attempted; this was done iteratively until only 28 bp remained, at which point the read was considered unmappable. This end trimming typically doubled or more the number of mappable reads.

Expression levels were calculated by adding, for each base pair, the number of times that base pair was encountered in the RNA-Seq data. For example, if 14 reads overlapped a particular base pair, that base pair would have an expression level of 14. The expression level of a gene was calculated as the mean expression level across the annotated ORF; these values were quantile normalized for comparisons between multiple conditions.

The mappability of genes was calculated because if a gene is in or near an area of the genome with high homology to another genomic area, it becomes impossible to assign the genomic origin of an RNA-Seq read stemming from one of the homologous areas. Mappability of genes was determined by creating, for the plus and minus strands, simulated 76-mer and 28-mer reads starting at each base pair in the annotated genome and processing these reads in the pipeline. (Note from above that reads may have been 28, 32, 36, Δ, 76-mers.) Perfectly mappable genes would thus have an expression level of 104, as all 76 of the 76-mer reads and all 28 of the 28-mer reads would have intersected every base pair of the gene. Genes were considered mappable if the mean expression level across the gene was 90% or more of that (94 reads).

Calling 3′ end tags was done as in Nagalakshmi *et al.* 2008, which is further described in Wang *et al.* 2009; three or more nongenomic A bases were required for each tag.

For a full explanation of the end-calling algorithm, refer to the software source code. In summary, for each annotated ORF the log2 expression levels for YPAD exponential growth and the other condition were retrieved and median normalized. The standard deviation of the expression level difference over the annotated ORF was calculated as the measure of signal noise, here called *n*, since the median-normalized expression of the annotated ORF should have been the same in both conditions. Ends were called as the first region—determined by both a 10-bp and 80-bp sliding window—which was expressed at a level of 3.5 times *n* or more away from (*i.e.*, above or below) the expression level in YPAD exponential growth, although not less than a fourfold difference in expression. This cutoff was chosen by manual inspection and represents a conservative cutoff level. Tests were then applied to make sure that the called end was sufficiently expressed, was not an annotated intron, and was in a mappable region of the yeast genome. Each differential end also had to be at least 40 bp long.

RNA-binding protein (RBP) motifs from (Riordan *et al.* 2011) were called by using the consensus motifs in Supporting Information, Table S1 as a standard text search algorithm, for example, their motif AAACACAW could be matched to either AAACACAA or AAACACAU; no probabilistic weighting of nucleotide combinations was performed.

Differential expression was determined using the DESeq software package (Anders and Huber 2010). Subsequent Gene Ontology (GO) (Ashburner *et al.* 2000) analyses were conducted using the GO::TermFinder software (Boyle *et al.* 2004).

3′ RACE on the *CDC19* gene was carried out using the RLM-RACE kit from Ambion, following all instructions therein. The 3′ RACE outer primer had sequence CACCGAAACCGTCGCTGCCT and the 3′ RACE inner primer had sequence TTTTCGAACAAAAGGCCAAG.

Luciferase assays were performed using firefly luciferase with *Renilla* luciferase as a control, as described previously (McNabb and Reed 2005). Instead of integrating the luciferase constructs, however, they were used on a plasmid, as described in (Chu *et al.* 2011). Plasmid inserts were produced by DNA 2.0, and sequences are provided in Table S8.

## Results

### Mapping expressed regions of the yeast genome in 18 different conditions

In this study, we used strand-specific RNA-Seq (Parkhomchuk *et al.* 2009) to analyze the transcriptional landscapes of yeast under 18 different environmental conditions, detailed in Table 1. Each condition was analyzed using two biological replicates, and one additional technical replicate was performed for exponential growth in YPAD medium. In total, 850,570,328 reads of 76-bp length were sequenced on an Illumina GA IIx [see (Shendure and Ji 2008) for platform details], of which 670,446,084 reads and 377,263 poly-adenylated end tags (a total of 41,663,340,382 bases) were uniquely mapped to the yeast genome using Bowtie (Langmead *et al.* 2009).

**Table 1:** The 18 conditions under which RNA-Seq was performed

Condition	Description	No. of Sequenced Reads
Exponential growth	YPAD medium	52,497,608
Salt	1 M NaCl for 45 min	29,363,375
DNA damage	1 mM MMS for 1 hr	29,427,647
Alpha factor	2.5 mM for 45 min; add another 50 μL to 25 mL yeast for another 30 min	54,107,345
Sorbitol	1 M sorbitol for 45 min	55,210,660
Oxidative stress	0.4 M H_2_O_2_ for 45 min	34,262,447
Heat shock	in 37° shaker for 1 hr	36,362,297
Stationary phase	18 d in 30° incubator	32,982,185
SC media	Synthetic complete medium	30,783,099
SC glycerol media	4% glycerol instead of glucose in SC medium	35,241,114
High calcium	10 mM calcium chloride medium	37,389,705
Low nitrogen	1/5 the normal amount of Yeast Nitrogen Base in YPAD	27,864,332
Low phosphate	see File S1	30,373,844
Calcofluor	0.1% for 1 hr	31,921,767
Hydroxyurea	0.075M for 1 hr	27,825,606
Grape juice	Walgreen’s brand grape juice, filtered	50,208,736
Benomyl	5 μg/mL for 1 hr	40,974,093
Congo red	30 μg/mL of Congo red for 1 hr	33,650,224

Unless otherwise noted, the conditions consist of YPAD medium with the indicated reagents added for the indicated incubation time. SC, synthetic complete.

**Figure 1:**
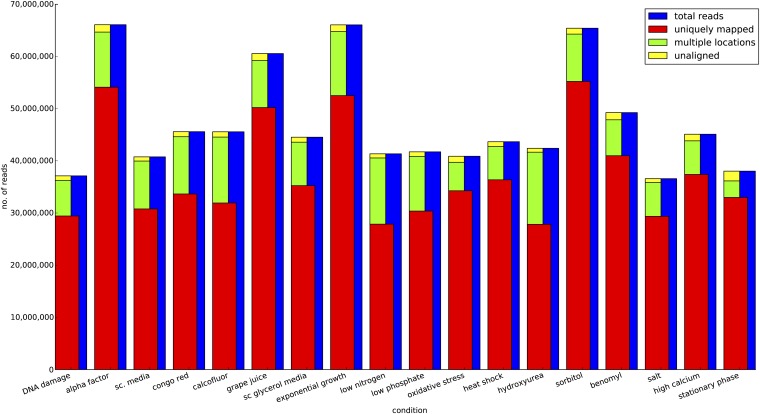
Sequencing done for each condition. Shown is the number of sequence tags generated for each condition (*i.e.*, two biological replicates, pooled). Of the reads labeled as “unaligned,” some still provide useful information as poly-A tags.

The number of uniquely mapped reads per condition ranged from 28 to 55 million (hydroxyurea and sorbitol, respectively), and the biological replicates showed high correlation. The Pearson *r* values of gene expression levels, calculated as described in the Methods section, ranged from 0.981 to 0.999 (alpha factor and high calcium, respectively). Figure 1 shows the number of reads sequenced for each condition, and Table S1 shows the Pearson correlations (*r* values) of the biological replicates for a given condition.

Approximately 93% of the yeast genome can be uniquely mapped with 76-bp reads. Most of the yeast genome is expressed, consistent with previous studies (*e.g.*, David *et al.* 2006; Nagalakshmi *et al.* 2008), and the data in this study demonstrate that approximately 80% (range 77–87%) of the base pairs in the yeast genome are expressed from one or both strands (*i.e.*, as a percentage of the 12-Mb genome) at a depth of five sequencing reads of coverage or more per base (Figure S1). At the same level of coverage, approximately 45% (range 40–51%) of the base pairs are expressed as a percentage of the 24-Mb genome, *i.e.*, separating out the Watson and Crick strands. Although we attempted to minimize potential artifacts from second-strand synthesis from reverse transcriptase, it is still possible that some of the observed transcription might be due to reverse transcription. The RNA-Seq method used in this study did not yield lower levels of antisense transcription when actinomycin D was used (Parkhomchuk *et al.* 2009), unlike that found in previous studies (Perocchi *et al.* 2007; Levin *et al.* 2010). Examination of annotated genes, however, revealed that transcription from one strand was accompanied by only a fraction of that amount of transcription occurring on the opposite strand. The median gene’s ratio of sense to anti-sense transcription was 5.1 to 6.4 log-two power (Figure S3). These results indicate that most of the yeast genome is truly expressed under different conditions and that some true antisense transcription is present, as expected (Xu *et al.* 2009).

### Most yeast genes are differentially expressed

We analyzed gene expression across the 18 conditions. Of 6058 mappable genes (*i.e.*, not in repetitive regions), we found that 5958 genes were expressed in at least one condition (with a threshold of each ORF base detected five or more times on average); 100 genes fell below this threshold, and 85 of these were classified as “dubious.” Interestingly, 611 dubious genes are expressed at or above our threshold.

Analysis of differential expression for the 5362 genes which are both mappable and nondubious was done using DESeq (Anders and Huber 2010). Using a binomial test (*P* ≤ 0.01), the majority (5245; 97.8%) was differentially expressed relative to exponential growth in YPAD media in at least one condition. Adding a criterion of 1.5-fold enrichment reduced this number to 4800 (89.5%), and this figure further reduces to 3421 (63.8%) when a 2-fold threshold is used. Interestingly, only 117 genes never differ in their overall RNA levels when we use the 1.5-efold enrichment threshold, listed in Table S9. These are enriched for the GO categories “biological process,” “intron homing,” “cellular component,” “molecular function,” and “endonuclease activity” (Ashburner *et al.* 2000) at a *P*-value of 0.05 (corrected) against a background set of mappable genes; the GO::TermFinder software suite (Boyle *et al.* 2004) was used to calculate enrichment.

GO-enrichment analysis of the genes that are differentially expressed using the binomial test revealed largely expected results, *e.g.*, alpha factor−stimulated cells had as their top five categories “conjugation,” “conjugation with cellular fusion,” “sexual reproduction,” “interaction between organisms,” and “response to pheromone.” Similarly, the top five categories for Congo red stimulation, which damages cell walls, were “cell wall organization and biogenesis,” “external encapsulating structure organization and biogenesis,” “cell morphogenesis,” “morphogenesis,” and “anatomical structure development”; one of the top categories for heat shocked cells was “protein folding” (see Table S2 for complete results).

Interestingly, cells grown in commercial grape juice had as their top GO category “vitamin metabolism,” and the 41 genes in this category contained many vitamin biosynthetic genes related to thiamine, nicotinamide adenine dinucleotide/nicotinamide adenine dinucleotide phosphate, flavin, and carnitine, and are aimed at rapidly processing sugar. Two genes among these, *PYC1* and *PYC2*, are associated with lactic acidosis when their human homolog is mutated [gene annotations taken from the Saccharomyces Genome Database (Cherry *et al.* 1998)].

### Extensive differences in 5′ ends in stress conditions

We were particularly interested in examining differential transcript end diversity in yeast. A custom software pipeline was developed for the identification of differentially expressed transcript ends. This pipeline compared signal tracks for the exponential growth condition to every other condition, one at a time, and called differential ends when the difference in signal between the two conditions exceeded a stringent threshold calculated for each gene based on internal signal noise (see *Materials and Methods*; Figure S4). As transcriptional landscapes were directly compared, the term “topology comparisons” was used to describe this approach. Table S3 contains the complete list of alternate ends.

**Figure 2:**
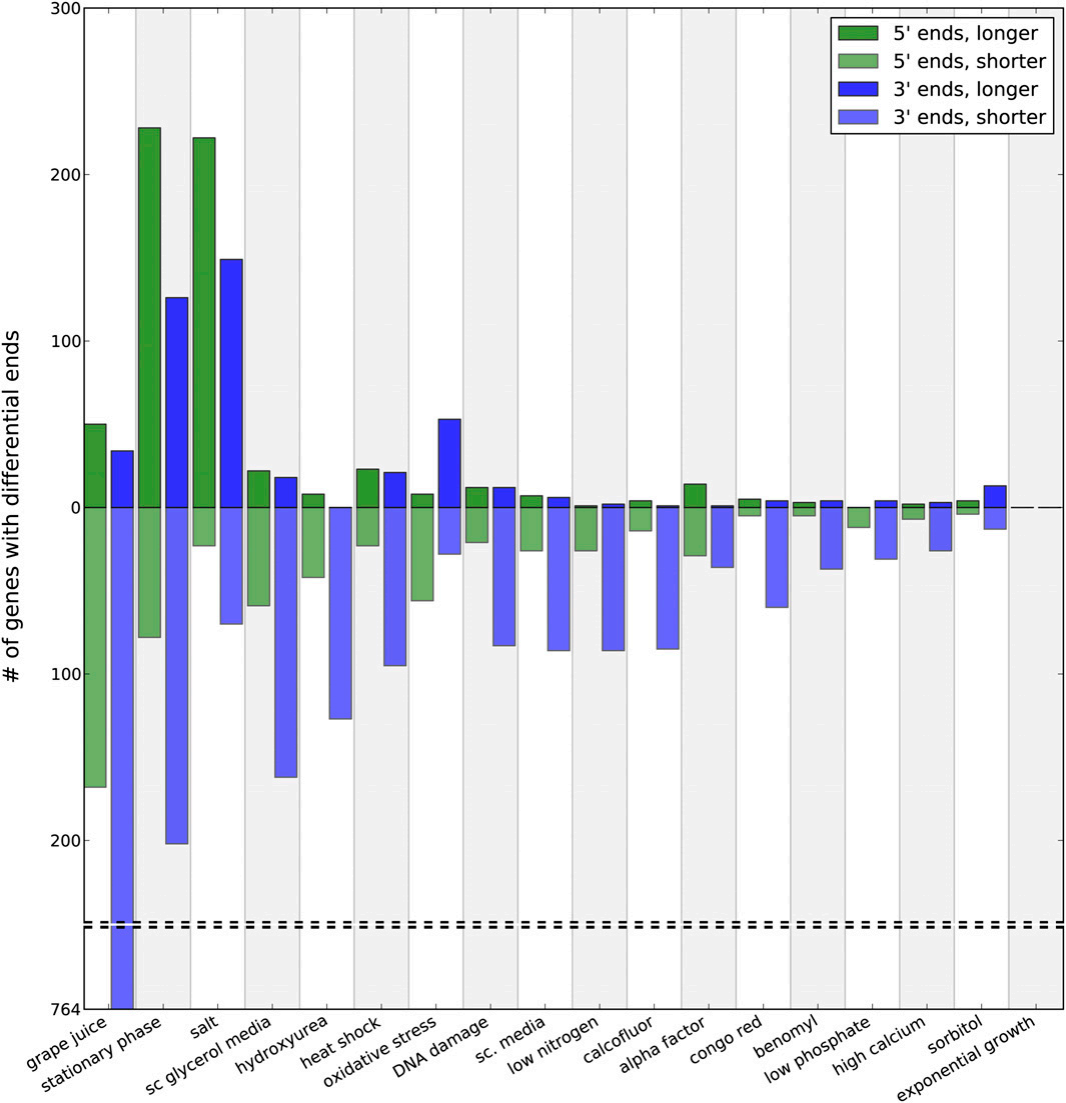
Number of differential ends. Shown is the number of genes with differential longer and shorter ends on either the 5′ or 3′ side. All comparisons were with the exponential growth condition.

Extensive differences were observed in 5′ and 3′ ends, and Figure 2 summarizes the number of genes with differential ends. Relative to cells grown in rich medium, we find 613 cases of longer 5′ ends and 598 instances of shorter 5′ ends across the conditions, affecting 471 and 364 unique ORFs, respectively. For some conditions (*e.g.*, alpha factor) cells exhibit minimal use of alternative 5′ ends, and, of those that do, the differential 5′ ends most often are shorter relative to cells grown in rich medium, that is, the transcription start site is closer to the start codon for translation for the nonrich medium condition.

Two conditions, salt stimulation and stationary phase, show an abundance of transcripts with increased 5′ UTR size, with more than 200 genes each expressing a longer variant of its transcript relative to growth in rich medium. Of 15 transcripts with an alternative 5′ UTR longer than 300 base pairs, seven were found in stationary phase and four under salt stimulation. The longest alternative 5′ UTR, for the gene *CDC8*, was 853 base pairs longer in cells in stationary phase.

One interesting effect is when a differential transcript is shortened to the point that the annotated start codon is not transcribed. This is observed in 21 instances and 18 unique genes across 10 conditions; the affected genes are *AAC1*, *ATG16*, *BRL1*, *CCM1*, *COS6*, *COX23*, *EMI2*, *FYV10*, *GEP7*, *GLR1*, *LAP2*, *MAD1*, *MRN1*, *MSN4*, *PRP39*, *RDH54*, *SUC2*, and *UBP7* (Table S1). Of these, only *SUC2* has been characterized previously as a gene that encodes two transcript forms that produce proteins with differential amino termini (Carlson and Botstein 1982).

### Upstream ORFs lie in many differential 5′ ends

Examining differential 5′ ends identified across all conditions revealed a total of 614 instances of uORFs in the differential transcribed region (see *Materials and Methods*). These affect 446 transcripts and a total of 543 unique uORFs (47 of which consist of solely a start and stop codon, with no intervening codon). These results are presented in Table S4.

We next compared the number of uORFs found in differential ends to the number expected by chance in differential ends of the same sizes, using random sampling of promoter sequences within 1000 bp of annotated start codons in the yeast genome. We found that for cells incubated in the presence of high salt, transcripts were statistically enriched for uORFs in the differential 5′ ends (z-value > 3). Interestingly, there was no statistical enrichment of uORFs in shortened 5′ UTRs in salt, but only in longer 5′ UTRs. Of the 222 genes with a longer alternative 5′ UTR in the salt response, 104 had one or more uORFs whereas a total of 148 uORFs were introduced in the longer-form UTR, where only 107 (std. dev.: 9.7) would have been expected by chance alone (Table S5). Overall, these results indicate that the differential 5′ ends, particularly long ones, are found under cell growth in extreme conditions such as high salt.

### Diverse roles of differential uORFs in salt response

**Figure 3:**
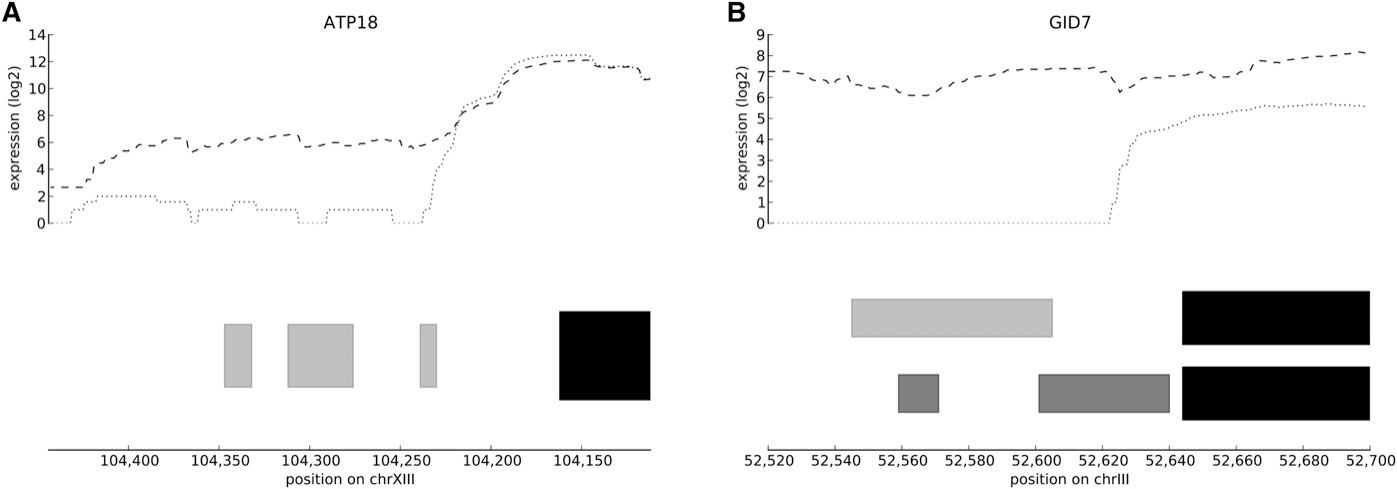
uORFs knockouts. Illustration of the manner in which uORFs were mutated. The upper track shows expression, the dotted line under exponential growth in YPAD, and the dashed line under salt stimulation conditions. The lower track shows the main ORF in solid black and the knocked-out uORFs in light gray. Out-of-register ORFs, which were not knocked out, are shown in dark gray. (A) *ATP18*; (B) *GID7*. In the case of *GID7*, the shorter of those two out-of-register ORFs is conserved across *Saccharomyces paradoxus*, *Saccharomyces mikatae*, and *Saccharomyces bayanus* (see sequence data from Cliften *et al.* 2003). Please also note that the exact insert placed into the plasmid ran from the far left of each panel to the beginning of the main ORF; the latter was replaced with luciferase.

**Figure 4:**
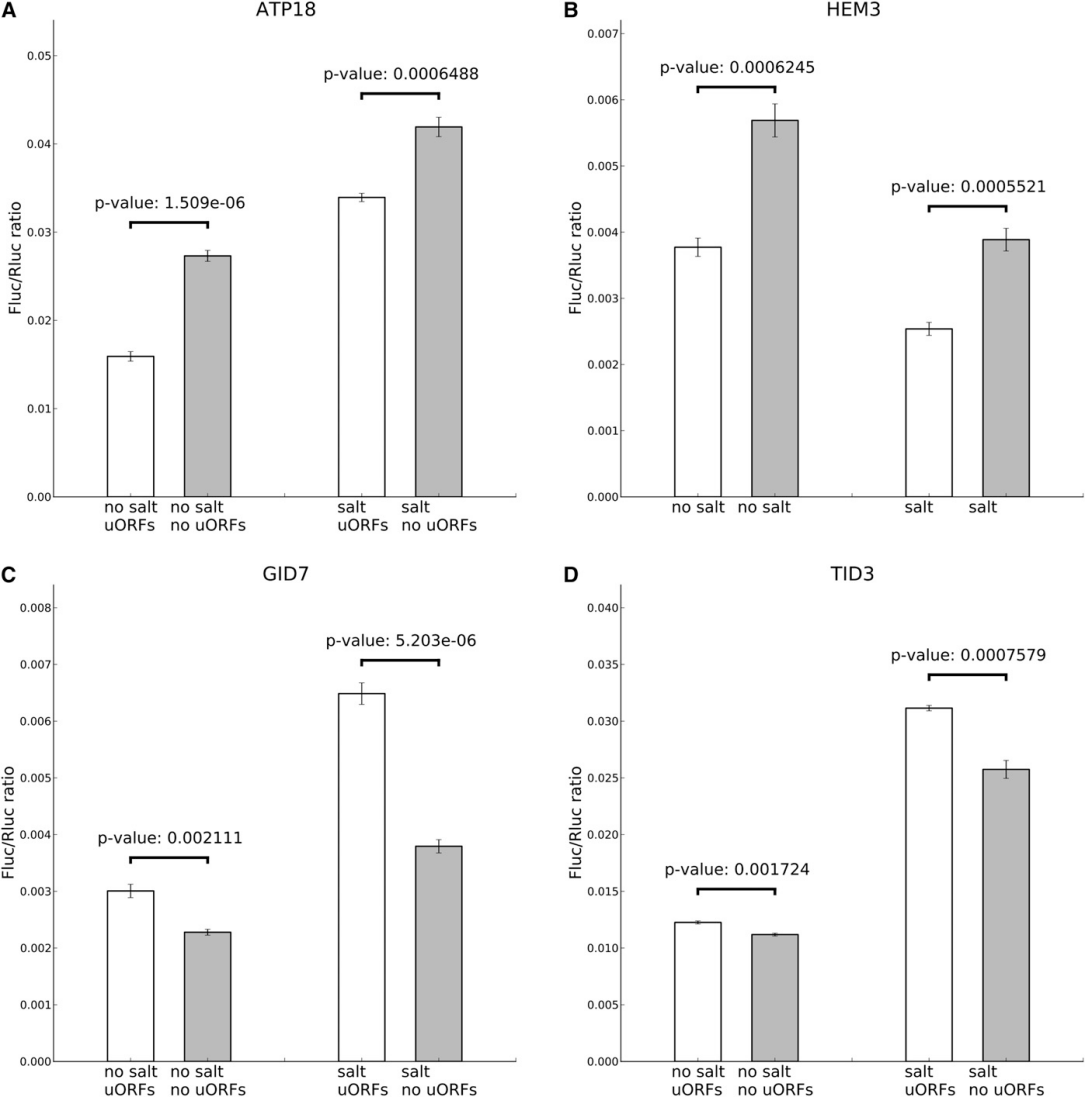
Luciferase analysis of uORFs. Luciferase assays confirming the relevance of uORFs in four genes with longer alternative 5′ UTRs in the salt stress condition. (A) ATP18; (B) HEM3; (C) GID7; and (D) TID3. The left two bars show results in SC–ura media and the right two bars show results in SC–ura media with 1 M salt for 1 hr. White bars are the results using the leader sequences, and gray bars have had uORF start codons mutated.

To further analyze the role of differential uORFs in gene expression, we selected five genes with uORFs for further study, and fused their upstream sequences from the gene’s start codon to the nearest neighboring gene upstream of a firefly luciferase reporter. A *Renilla* luciferase gene fused to a constitutive promoter was used as a loading control for these same cells (McNabb and Reed 2005; plasmid pTH650 described in Chu *et al.* 2011). A version of the upstream sequence in which the “in register uORFs” had been removed also was produced based on sequential AUGs, where the AUG start codon was changed to UUG (see Figure 3 for an explanation). In each case, the longest uORFs and the first accessible uORFs were removed. Results of the luciferase assays are presented in Figure 4.

Of note, *HEM3* and *ATP18* were expressed less in salt relative to YPAD according to the RNA-Seq data (0.6× and 0.5×, respectively), and the uORFs were found to play a repressive role, *i.e.*, the mutated, uORF-less construct was expressed greater. *GID7* and *TID3*, however, were expressed greater in salt (2.5× more in both cases), and the plasmids with uORF knockouts express less reporter indicating that the uORFs for these genes played an activating role in protein expressed. *RPH1* also was examined, although mutations analyses revealed no change indicating that its uORFs did not appear to play a significant role controlling gene expression.

As a further control that the uORFs themselves were responsible for the translation changes and not a condition-specific splicing event (*e.g.*, Cheah *et al.* 2007). TopHat (Trapnell *et al.* 2009) was used to analyze the transcripts for splicing events. No evidence of alternative splicing was found. Thus, we conclude that most uORFs affect gene expression; for some genes they activate gene expression and in other instances they repress gene expression

### Extensive differences in mRNA 3′ ends

Analysis of 3′ ends revealed a number of genes with differential 3′ ends in each of the various conditions as determined by topology comparisons (Figure 2). Relative to exponential growth in YPAD, there were 451 instances of longer 3′ ends, and 1991 instances of shorter 3′ ends across all conditions, representing 328 and 993 unique ORFs, respectively. Table S3 contains the complete list of altered 3′ lengths. Yeast grown in grape juice was clearly distinct, with 764 genes having a shorter transcript. Interestingly, one gene, *REE1*, was shortened in every condition relative to rich medium.

In addition to topology comparisons, analysis of 377,263 poly-adenylated end tags in our data set can be used to analyze 3′ ends. These are sequencing reads with a nongenomic run of A bases at the ends of mappable sequences are presumed to be a poly-adenylation event and thus mark the end of a transcript. These reads are recovered from the population of reads that did not initially map to the yeast genome, as the poly-adenylated tail will prevent alignment. Determining differential ends using this approach from our sequencing method is sensitive to thresholding and vulnerable to noise from low sampling levels; with 377,263 such reads across 18 conditions and a 6000 gene organism, only 3.5 reads per gene would be expected in each condition, assuming a uniform distribution of gene expression levels. In addition, poly-adenylated tags may be hampered by artifacts due to oligo-dT primer ligation to A-rich genomic sequences. Topology comparisons are thus relied upon as the main approach in this study.

Ozsolak *et al.* (Ozsolak *et al.* 2010) used poly-adenylation to call transcript ends because their protocol only sequenced poly-adenylated regions of the transcriptome and does not suffer from undersampling. They found 91,891 poly-adenylation sites (File S1), and reported that 72.1% of yeast genes have at least two poly-adenylation sites further than 50 bp apart.

**Figure 5:**
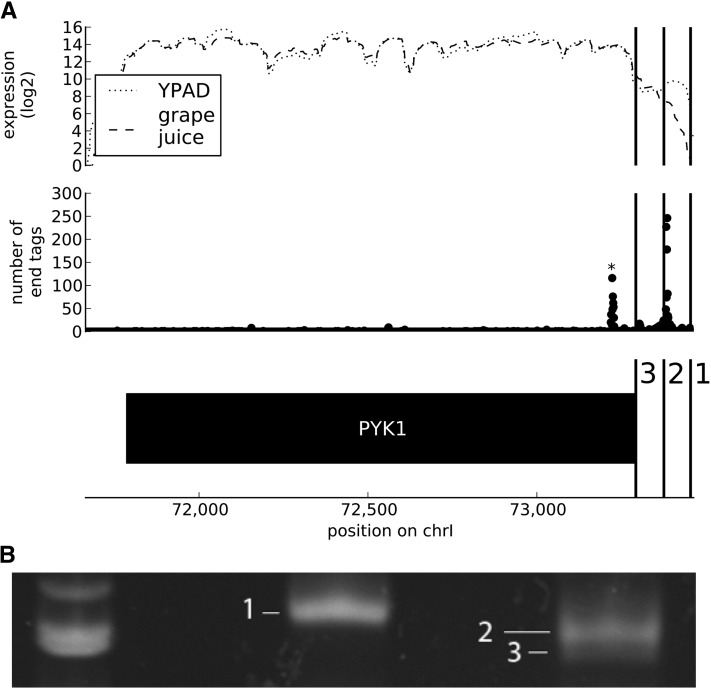
Pyruvate kinase differential end validation. (A) The top track shows the expression profile for *CDC19*/*PYK1* (pyruvate kinase) under exponential growth in YPAD and grape juice conditions. The middle track shows the number of sequenced end tags from the study as a whole. The bottom track shows the annotated ORF. The numbers 1, 2, and 3 show Sanger sequencing results from the 3′ RACE bands in part B. (B) Gel showing the results of 3′ RACE PCR for *CDC19*. The ladder shows the 600- and 500-bp markers. The center lane is the result for RNA from the exponential growth condition, and the right lane is from grape juice. The bands labeled 1, 2, and 3 were sequenced and correspond to the marked locations in part A.

Pooling the results for all conditions in our RNA-Seq data, we found that 171 genes had more than 10 end tags. Of those, 128 (74.9%) had two or more discrete poly-adenylation start sites, indicating variability in 3′ end structure. Among our interesting results, we found one gene, *CDC19*/*PYK1* (pyruvate kinase), which has an alternative 3′ end (Figure 5). Rapid amplification of cDNA ends polymerase chain reaction (RACE PCR) confirmed the existence of three distinct ends, one of which is visible only as an expression drop, another only from poly-adenylation tags, and a third visible from both. In a study of 3′ UTRs in *Caenorhabditis elegans* using polyA capture, 3′ RACE, cDNA sequencing, and RNA-Seq, 27% of called ends were only called using one technique (Mangone *et al.* 2010); thus, the use of multiple methods for calling ends is generally useful for 3′ end mapping.

Although the effects were often less dramatic than those seen at the 5′ end, expression at the 3′ end can decrease to levels where the main stop codon is not part of the dominant form of the transcript, or not present at all in the data. Figure S2B shows the 3′ end of the *VMA5* gene, which does not appear to have a stop codon at all in synthetic complete media. Under exponential growth, there are 32 poly-adenylated end tags near *VMA5* between genomic co-ordinates 285,831 and 285,837 on chromosome XI, although there is clearly some expression past that point, which would expose the annotated stop codon in a minority of transcripts.

In another example, *PPZ2*, which encodes a phosphatase involved in regulating cell cycle progression and shown in Figure S2A, the transcript level for the gene as a whole increases by 2.2-fold in the shift from exponential growth to stationary phase, but the level of the differential end, called algorithmically from genomic co-ordinates 1,336,923 to 1,336,988 on chromosome IV, increases by 15.8-fold (after we adjusted for sequencing library size). A total of 97 other instances of a shortened 3′ end were found using topology comparisons, represented in 63 unique ORFs in examples from all 17 non-exponential growth conditions. They are listed in the Table S6. Thus, many yeast transcripts may lack a translation termination codon.

The differential ends called by topology comparisons were examined for RBP motifs using the motifs published in (Riordan *et al.* 2011). Most conditions were statistically enriched for RBP motifs among the 3′ sequences that were shortened relative to rich YPD medium, with the exception of the oxidative stress and sorbitol conditions (Table S7). However, only five conditions were enriched for RBPs among the lengthened 3′ sequences (grape juice, oxidative stress, heat shock, sorbitol, and stationary phase). Statistical enrichment was calculated, similarly to that of uORFs, based on random sampling from the yeast genome of sequences of equal length to the differential ends, and a z-value cutoff greater than 3. Thus, transcripts with differential ends are likely to affect differences in RNA binding proteins and presumably gene regulation.

## Discussion

### Prematurely terminated transcripts

One particularly surprising finding of our study is that 63 ORFs were found which, under at least one condition, showed a 3′ end that likely terminated before the annotated stop codon. These transcripts would be left without a stop codon, potentially triggering a nonstop decay of the mRNA (Frischmeyer *et al.* 2002) and/or production of a polylysine tail on the protein that is produced. *PPZ2*, whose transcript exhibits this property, is a serine/threonine phosphatase involved in regulating cell-cycle progression, so its appearance in the stationary phase condition is of interest. The phenomenon of premature poly-adenylation has been noted before and described as an attenuating regulator of human mobile elements (Perepelitsa-Belancio and Deininger 2003), and the nonstop decay pathway has been described in yeast (Frischmeyer *et al.* 2002). Although the pathway is understood, there is little understanding of how prevalent it is (Garneau *et al.* 2007). This study indicates the particular genes that may be controlled in this fashion and that the phenomenon made be more widespread that previously appreciated.

### 5′ ORF truncations

Of the 18 genes found to have 5′ ORF truncations, *i.e.*, the dominant form of the transcript did not contain the annotated start codon, one, *SUC2*, has been extensively characterized previously. For *SUC2* the long form contains the signal sequence that causes the sucrose invertase to be secreted (Carlson and Botstein 1982). Carlson and Botstein found that glucose-repressed cells produce a longer form of *SUC2*, which is consistent with the results of our study, in which the RNA-Seq data show a longer transcript present in media using glycerol as the carbon source. In addition to *SUC2*, 17 other genes produce shorter proteins. Only *SUC2*, however, is predicted to encode a protein that contains a signal sequence that is missing in the shorter transcript [performed using SignalP 4.0 (Petersen *et al.* 2011)]. The alternative amino terminal sequence in these 17 instances may affect protein function in other ways.

### RBP motifs

In 2442 differential 3′ ends (1991 shortened and 451 lengthened) across 17 non-YPAD exponential growth conditions, 6420 RBP motifs were present (motifs from Riordan *et al.* 2011). RBP motifs are, to date, quite degenerate in the method they are defined, and this likely leads to many false positives. Nevertheless, statistical enrichment was more often seen in shortened 3′ ends, suggesting that cells may remove the layer of control offered by RBPs under stress conditions.

### uORFs

Upstream ORFs are a known regulatory feature. The availability of genomic sequences for many organisms, yeast since 1996 (Goffeau *et al.* 1996), has allowed for a theoretical search for uORFs, and a large abundance has been found. Whether they have meaning as a method for translational control depends first and foremost on whether they are transcribed; these data have been harder to produce. Using a random lacZ insertion method, Burns *et al.* and Ross-Macdonald *et al.* revealed extensive translation of short and out of frame ORF expression through the yeast genome (Burns *et al.* 1994; Ross-Macdonald *et al.* 1999). In a recent RNA-Seq study, Nagalakshmi *et al.* (2008) concluded that 6% of genes had uORFs. Estimates vary and range up to 20% (Hood *et al.* 2009). Our data indicate that many of these lie in differential UTRs.

Two broad mechanisms of uORF action are known (see Vilela and MCcarthy 2003): control by uORFs modulating posttermination behavior of ribosomes and control modulated by the actual peptide produced by a uORF. The canonical example of the first is *GCN4*, characterized in (Mueller and Hinnebusch 1986), and of the second the arginine attenuator peptide found before the *CPA1* gene (Gaba *et al.* 2001).

Even if transcribed, whether uORFs have regulatory function further depends on their interaction with the translational machinery. Genes such as *GCN4* (Mueller and Hinnebusch 1986), *CPA1* (Gaba *et al.* 2001), and *YAP1*/*YAP2* (Vilela *et al.* 1998) are well characterized, and further examples do exist. Some, however, show no effect [see (Zhang and Dietrich 2005) where not all validate], and some show an effect dependent on RNA processing [see (Zhang and Dietrich 2005), for an example involving riboswitches in *N. crassa*]. Most studies to date have used bioinformatic approaches solely and analyze sequence context and/or conservation, which does not offer proof of uORF relevance [see *e.g.*, (Cvijović *et al.* 2007; Lawless *et al.* 2009; Selpi *et al.* 2009), all in *S. cerevisiae*]. In our study, in which we performed luciferase validation experiments, we examine five leader sequences with uORFs, only four of which had a direct impact on luciferase levels (Zhang and Dietrich 2005). In another study, authors analyzed the ribosome footprint to mRNA ratios in meiosis and concluded that uORFs in differential ends could alter translational efficiency of mRNA. They also reported that in the case of uORFs with an AUG start codon, the translational efficiency and ribosome occupancy showed a “strong negative correlation” (Brar *et al.* 2012). A subsequent microarray-based study of meiotic yeast confirmed the presence of abundant transcript architecture changes in meiotic and sporulating yeast (Kim Guisbert *et al.* 2012).

In this study, there was only a statistical enrichment for uORFs relative to promoter sequences in longer 5′ ends in the salt condition. Under no conditions was a statistically significant enrichment of uORFs present in shortened 5′ UTRs, implying that normally used uORFs are seldom removed by alternative promoter choices.

In each of the four cases of a uORF affecting translation reported in (Zhang and Dietrich 2005), removing the uORFs led to increased luciferase activity. In this study, a new phenomenon is reported: uORFs which increase luciferase activity (*GID7* and *TID3*; see Figure 4). That uORFs might increase translational efficiency has been hypothesized (Neafsey and Galagan 2007; see also Hood *et al.* 2009), but no previous examples have been found or directed demonstrated using mutational analyses. One possibly is that these uORFs have RNA binding sites or ribosome entry sites that attracting ribosomes to those mRNAs.

Overall, this study demonstrates pervasive differential transcript ends occur in yeast and that in a least several cases these differences likely affect gene expression. The mechanisms by which differential ends are selected remains to be determined, and such information will be important to obtain a details understanding of how eukaryotic gene expression is controlled.

## Supplementary Material

Supporting Information

RNA-seq data
