# Supplementary material for: Extensive Transcript Diversity and Novel Upstream Open Reading Frame Regulation in Yeast
Source: G3 (Bethesda). 2013 Feb 1;3(2):343–52. doi: 10.1534/g3.112.003640 (PMC3564994; doi:10.1534/g3.112.003640)
Supplement: Supporting Information [file supp_3_2_343__index.html]

Supporting Information 

# Extensive Transcript Diversity and Novel Upstream Open Reading Frame Regulation in Yeast

## Supporting Information for Waern and Snyder, 2013

**Files in this Data Supplement:**

- Supporting Information - Figures S1-S4, File S1, and Tables S1-S9 (PDF, 4.4 MB)
- Figure S1 - Read coverage of the yeast genome (PDF, 533 KB)
- Figure S2 - Two example 3' truncations (PDF, 2.5 MB)
- Figure S3 - Antisense transcription (PDF, 511 KB)
- Figure S4 - Topology comparisons method explanation (PDF, 926 KB)
- File S1 - Supporting Materials (PDF, 48 KB)
- Table S1 - Shows the correlation between the biological replicates done for each condition (PDF, 56 KB)
- Table S2 - (.xls, 8.2 MB)
- Table S3 - (.xls, 471 KB)
- Table S4 - (.xlsx, 47 KB)
- Table S5 - (.xlsx, 41 KB)
- Table S6 - (.xls, 48 KB)
- Table S7 - (.xlsx, 37 KB)
- Table S8 - (.xls, 22 KB)
- Table S9 - (.xls, 43 KB)
