# Supplementary material for: Extensive Transcript Diversity and Novel Upstream Open Reading Frame Regulation in Yeast
Source: G3 (Bethesda). 2013 Feb 1;3(2):343–52. doi: 10.1534/g3.112.003640 (PMC3564994; doi:10.1534/g3.112.003640)
Supplement: Supporting Information [file supp_3.2.343_003640SI.pdf]

G3: Genes | Genomes | Genetics

Extensive Transcript Diversity and Novel Upstream ORF Regulation in Yeast

Karl Waern [*,§] and Michael Snyder [*,§]

[*] Department of Genetics, Stanford University School of Medicine, Stanford, California 94305, United States of America

[§] Department of Molecular, Cellular, and Developmental Biology, Yale University, New Haven, Connecticut 06520, United States of America.

DOI: 10.1534/g3.112.003640

File S1

Supporting Materials

Description of low phosphate medium:

Low Phosphate YPD (LP-YPD) Medium

1. Combine: 5 g Yeast Extract

10 g Peptone

1.23 g $MgSO_4$

475 ml dH2O

2. Stir until completely dissolved (>15 minutes).

3. With vigorous stirring, slowly add 4 ml of concentrated NH_4OH dropwise.

4. Remove from stir plate. Allow salts to precipitate for ≥30 minutes at room temperature.

5. Vacuum filter with 1.2 µm filter circle (or gravity filter with Whatman #1 paper).

6. Vacuum filter with 0.22 µm filter circle (or gravity filter with Whatman #2 paper).

7. Add 10 g dextrose (or galactose) and dissolve.

8. Adjust to pH 6.5 - 7.0 with 6 M HCl.

9. Autoclave or filter sterilize. Store at 4°C.

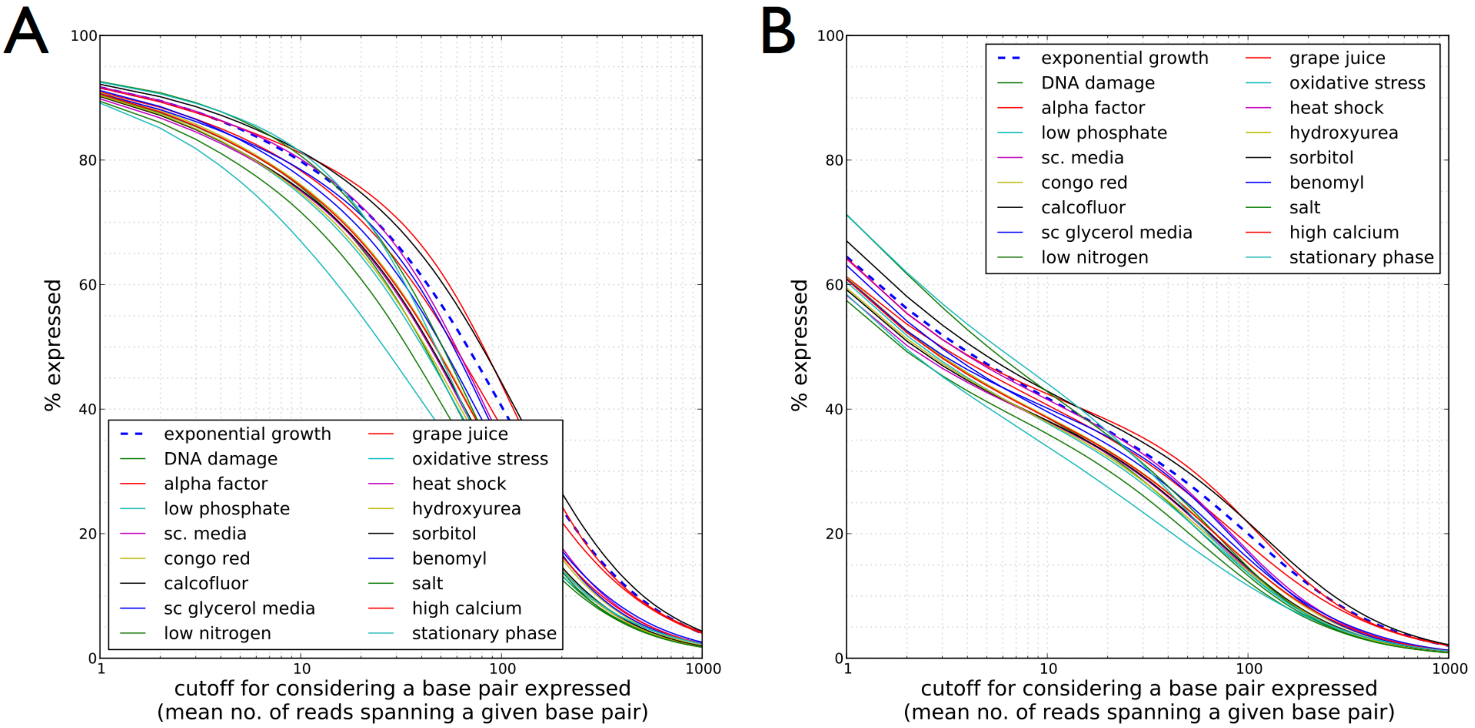

Figure S1 Read coverage of the yeast genome. Shows the percentage of the genome covered to a given read depth (or more). On the left are the percentages for transcription coming off either the plus or minus strand at any given locus, and the right shows percentages for the 24 Mb genome (*i.e.* treating the plus and minus strand separately).

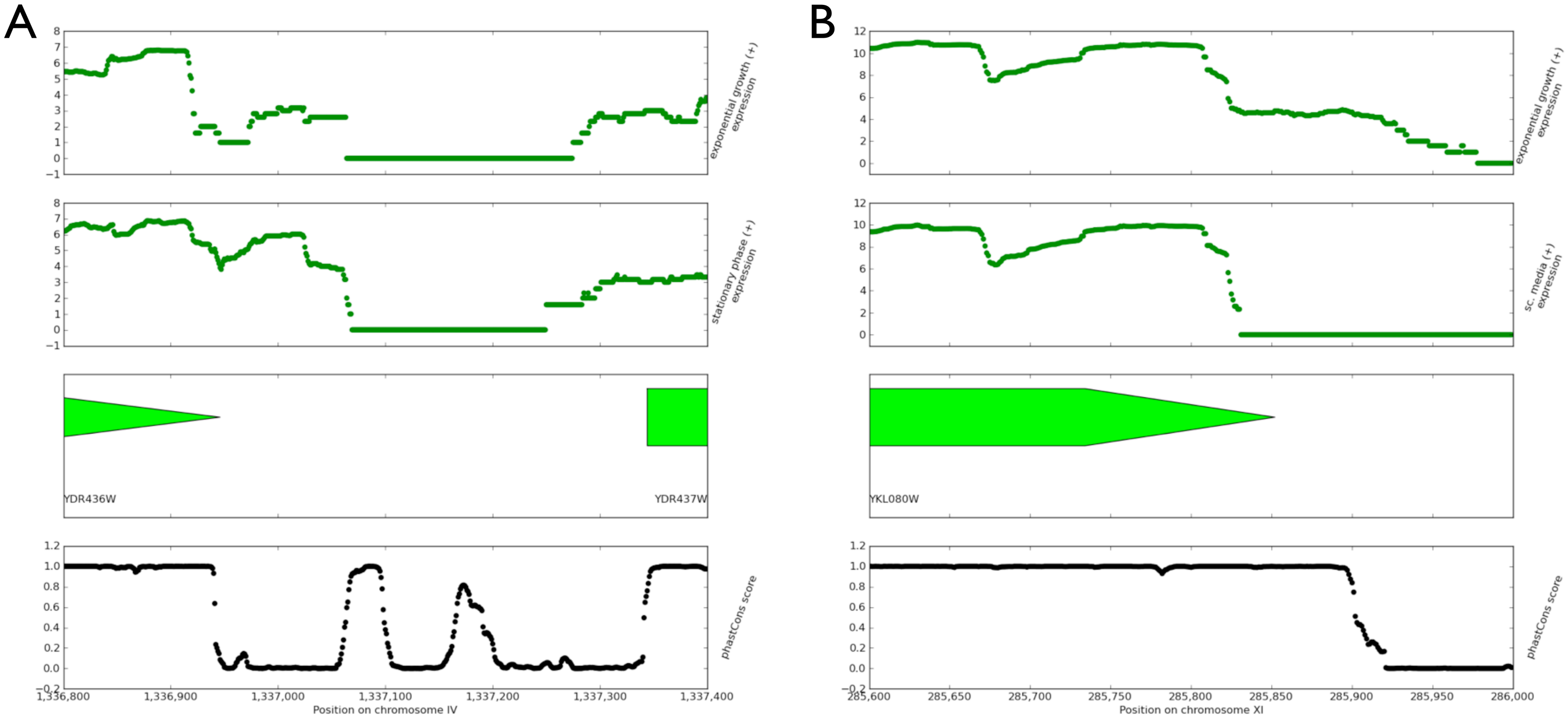

Figure S2 Two example 3' truncations. A: A close-up of the 3' end of the *PPZ2* gene. Note the log scale of the expression data. B: A close-up of the 3' end of the *VMA5* gene. In each case, the top track shows expression under the exponential growth in YPAD condition; the second track shows expression under the stationary phase condition; the third track shows the location of annotated ORFs; and the fourth track shows the phastCons conservation score (Siepel et al. 2005).

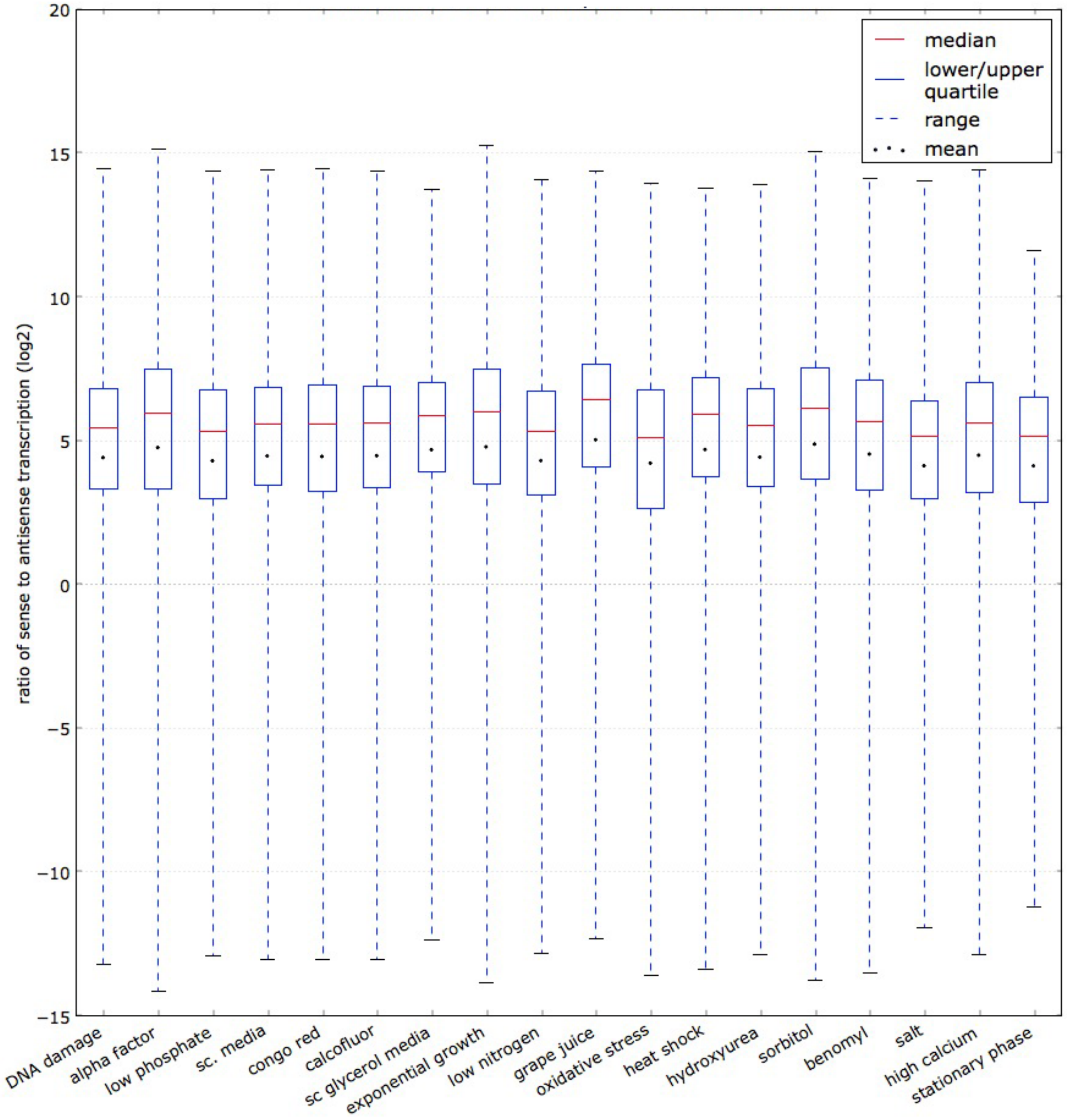

Figure S3 Antisense transcription. Shows average (mean and median) antisense transcription levels across all ORFs.

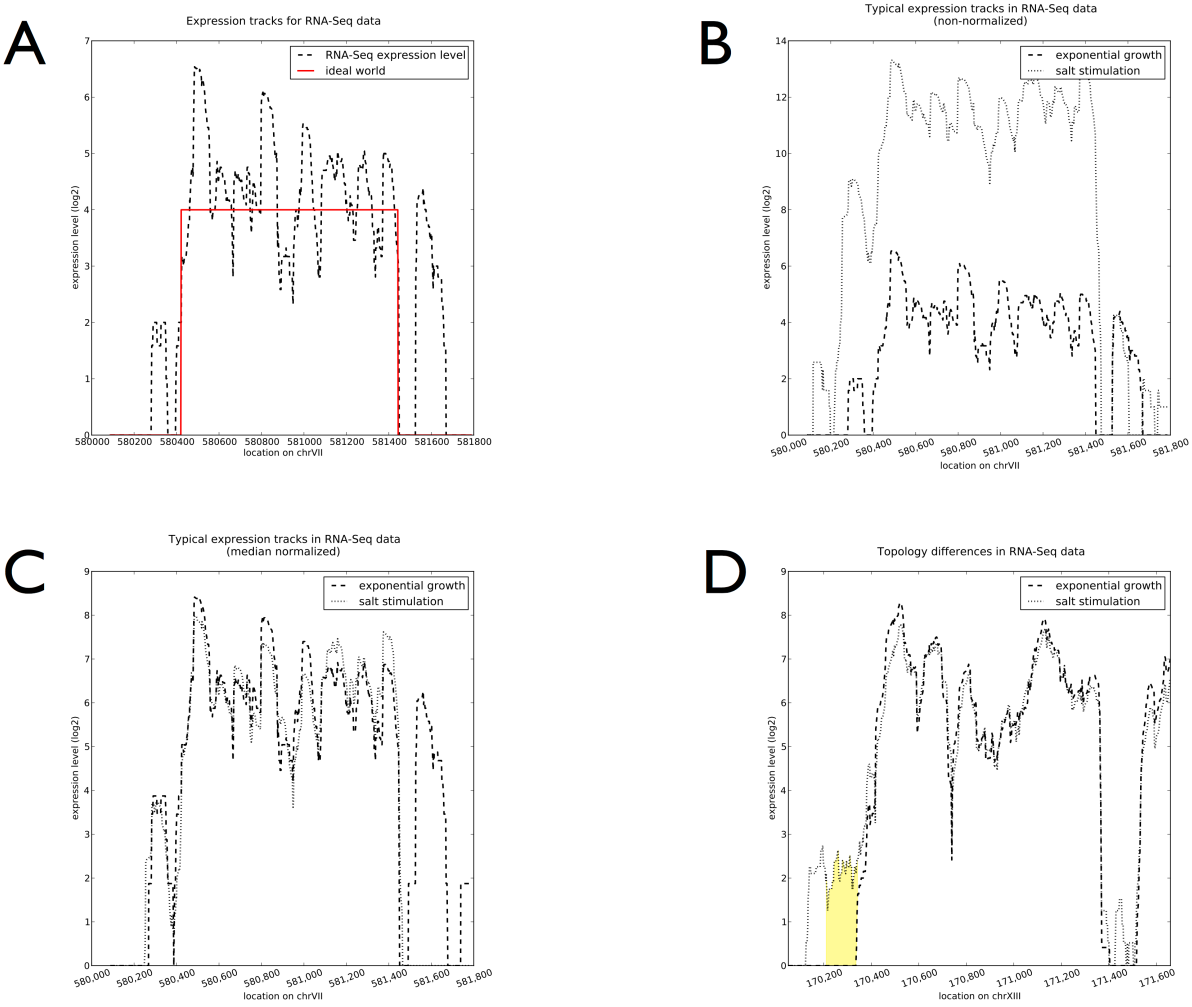

Figure S4 Topology comparisons method explanation. A: A sample RNA-Seq expression profile. The 'ideal world' line shows expression of the ORF with zero noise. B: Sample RNA-Seq tracks for the same ORF with expression under exponential growth in YPAD medium and under salt stimulation. Difference in height is due to either genuine expression level differences or library size differences. C: Median normalizing the two expression tracks from C shows that, while there is noise, the noise is very regular. D: An example of two expression tracks where there is a genuine topological difference after median normalization.

Table S1 Shows the correlation between the biological replicates done for each condition.

Condition	Pearson *r* Value
DNA damage	0.997
alpha factor	0.981
sc. media	0.996
congo red	0.997
calcofluor	0.994
grape juice	0.99
sc glycerol media	0.996
exponential growth	0.995
low nitrogen	0.995
low phosphate	0.994
oxidative stress	0.993
heat shock	0.993
hydroxyurea	0.998
sorbitol	0.996
benomyl	0.995
salt	0.991
high calcium	0.999
stationary phase	0.994

Tables S2 – S9

Available for download at http://www.g3journal.org/lookup/suppl/doi:10.1534/g3.112.003640/-/DC1.

.
