# Supplementary material for: Extensive Transcript Diversity and Novel Upstream Open Reading Frame Regulation in Yeast
Source: G3 (Bethesda). 2013 Feb 1;3(2):343–52. doi: 10.1534/g3.112.003640 (PMC3564994; doi:10.1534/g3.112.003640)
Supplement: Supporting Information [file supp_3.2.343_FigureS1.pdf]

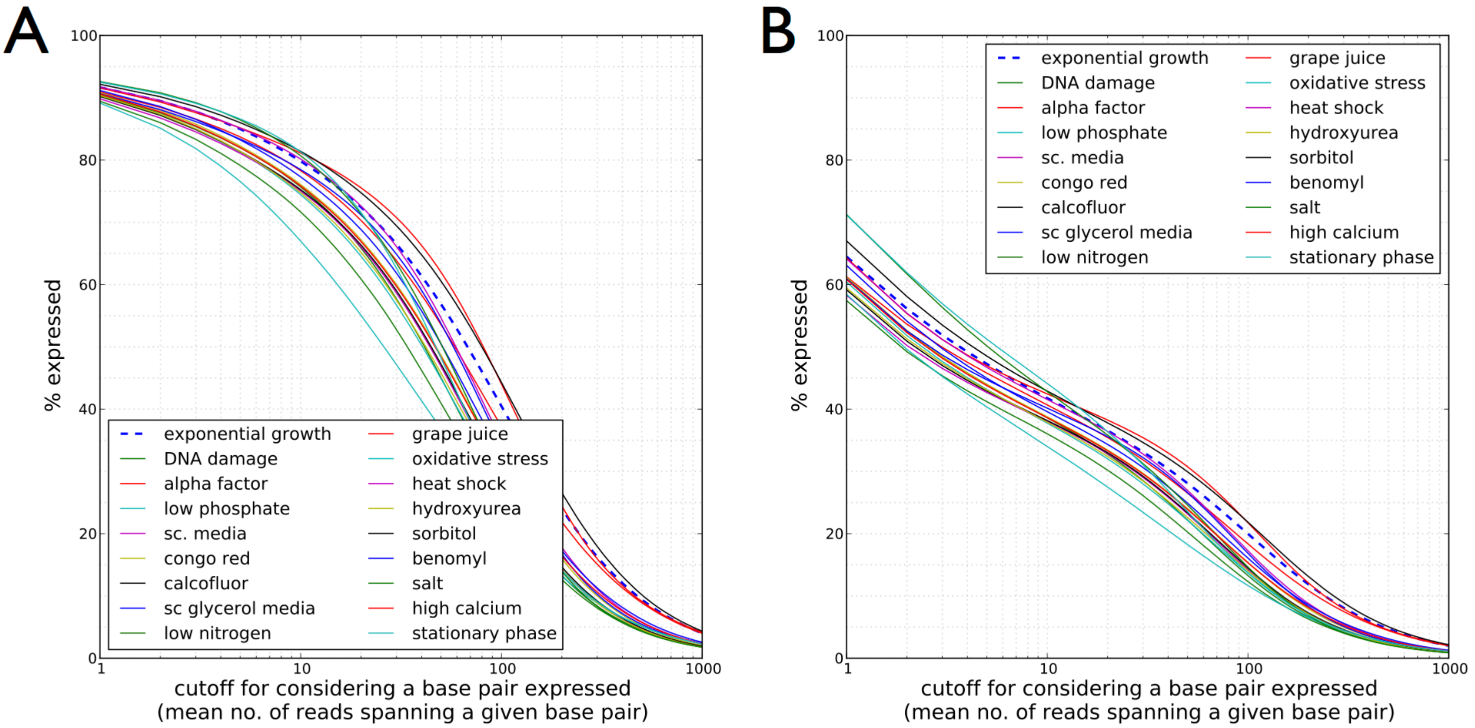

Figure S1 Read coverage of the yeast genome. Shows the percentage of the genome covered to a given read depth (or more). On the left are the percentages for transcription coming off either the plus or minus strand at any given locus, and the right shows percentages for the 24 Mb genome (*i.e.* treating the plus and minus strand separately).
