# Supplementary material for: Extensive Transcript Diversity and Novel Upstream Open Reading Frame Regulation in Yeast
Source: G3 (Bethesda). 2013 Feb 1;3(2):343–52. doi: 10.1534/g3.112.003640 (PMC3564994; doi:10.1534/g3.112.003640)
Supplement: Supporting Information [file supp_3.2.343_FigureS2.pdf]

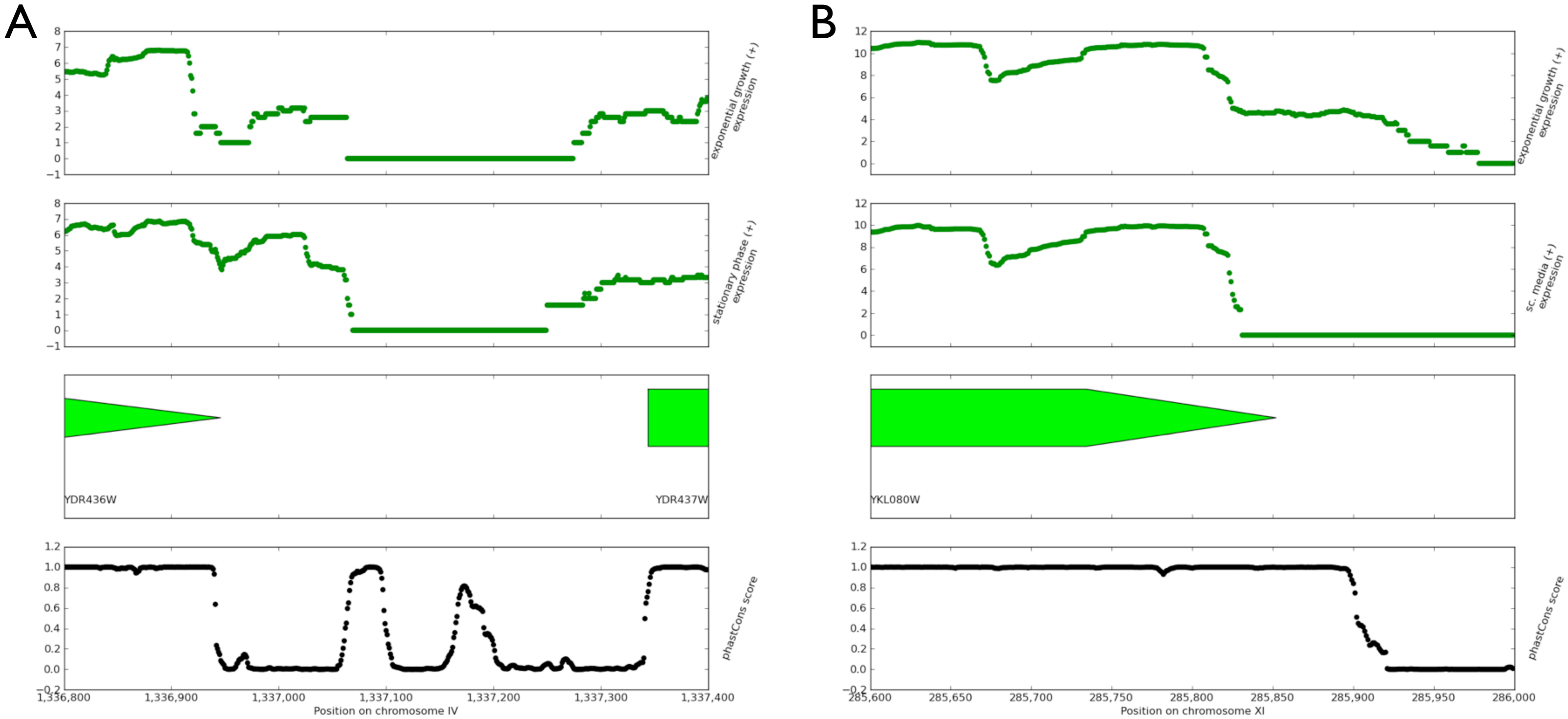

Figure S2 Two example 3’ truncations. A: A close-up of the 3' end of the *PPZ2* gene. Note the log scale of the expression data. B: A close-up of the 3' end of the *VMA5* gene. In each case, the top track shows expression under the exponential growth in YPAD condition; the second track shows expression under the stationary phase condition; the third track shows the location of annotated ORFs; and the fourth track shows the phastCons conservation score (Siepel et al. 2005).
