# Supplementary material for: Extensive Transcript Diversity and Novel Upstream Open Reading Frame Regulation in Yeast
Source: G3 (Bethesda). 2013 Feb 1;3(2):343–52. doi: 10.1534/g3.112.003640 (PMC3564994; doi:10.1534/g3.112.003640)
Supplement: Supporting Information [file supp_3.2.343_FigureS4.pdf]

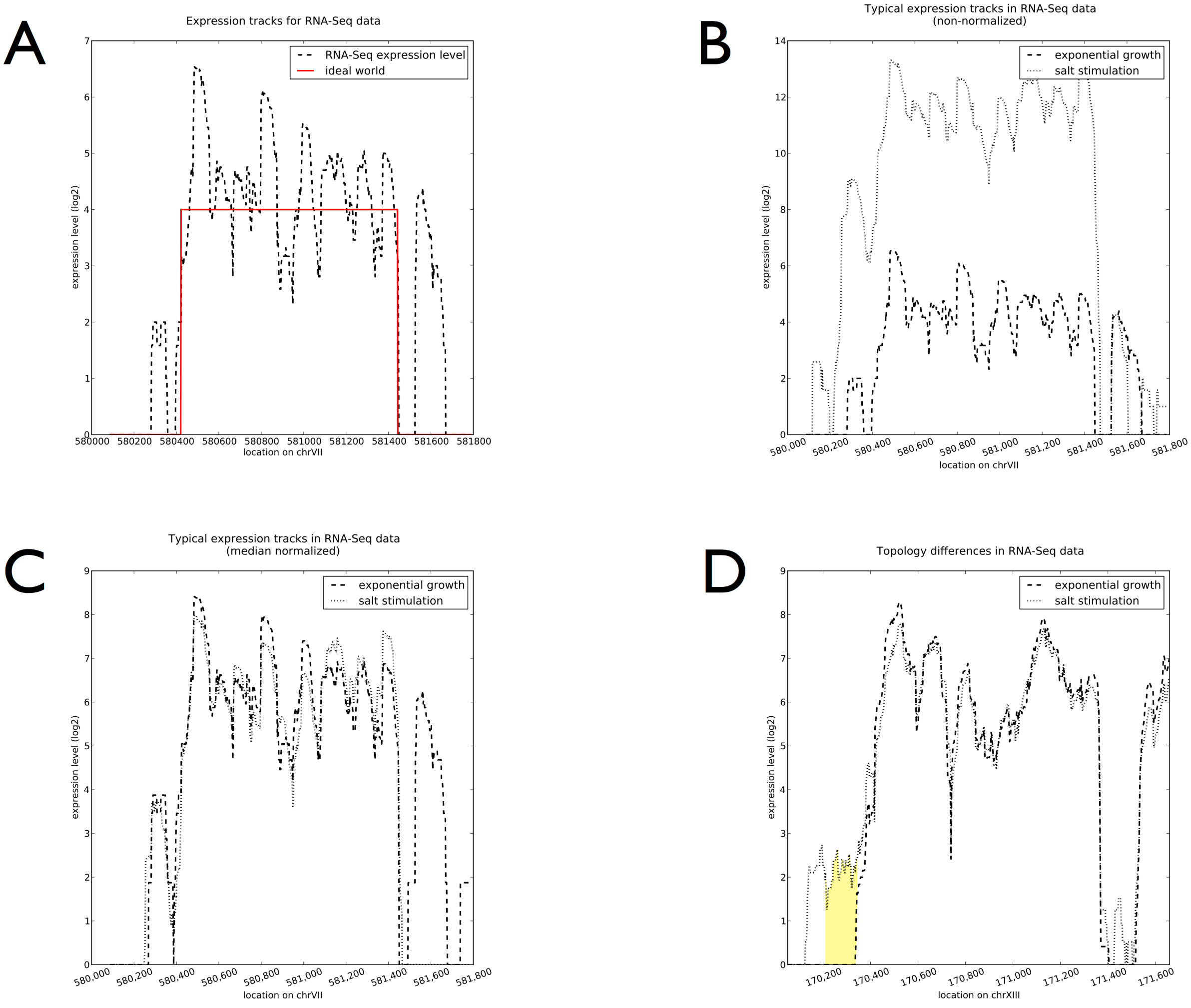

Figure S4 Topology comparisons method explanation. A: A sample RNA-Seq expression profile. The 'ideal world' line shows expression of the ORF with zero noise. B: Sample RNA-Seq tracks for the same ORF with expression under exponential growth in YPAD medium and under salt stimulation. Difference in height is due to either genuine expression level differences or library size differences. C: Median normalizing the two expression tracks from C shows that, while there is noise, the noise is very regular. D: An example of two expression tracks where there is a genuine topological difference after median normalization.
