# Supplementary material for: Extensive Transcript Diversity and Novel Upstream Open Reading Frame Regulation in Yeast
Source: G3 (Bethesda). 2013 Feb 1;3(2):343–52. doi: 10.1534/g3.112.003640 (PMC3564994; doi:10.1534/g3.112.003640)
Supplement: Supporting Information [file supp_3.2.343_FileS1.pdf]

File S1

Supporting Materials

Description of low phosphate medium:

Low Phosphate YPD (LP-YPD) Medium

1. Combine: 5 g Yeast Extract

10 g Peptone

1.23 g $MgSO_4$

475 ml dH2O

2. Stir until completely dissolved (>15 minutes).

3. With vigorous stirring, slowly add 4 ml of concentrated NH_4OH dropwise.

4. Remove from stir plate. Allow salts to precipitate for ≥30 minutes at room temperature.

5. Vacuum filter with 1.2 µm filter circle (or gravity filter with Whatman #1 paper).

6. Vacuum filter with 0.22 µm filter circle (or gravity filter with Whatman #2 paper).

7. Add 10 g dextrose (or galactose) and dissolve.

8. Adjust to pH 6.5 - 7.0 with 6 M HCl.

9. Autoclave or filter sterilize. Store at 4°C.
