# Supplementary material for: Extensive Transcript Diversity and Novel Upstream Open Reading Frame Regulation in Yeast
Source: G3 (Bethesda). 2013 Feb 1;3(2):343–52. doi: 10.1534/g3.112.003640 (PMC3564994; doi:10.1534/g3.112.003640)
Supplement: Supporting Information [file supp_3.2.343_TableS1.pdf]

Table S1 Shows the correlation between the biological replicates done for each condition.

Condition	Pearson *r* Value
DNA damage	0.997
alpha factor	0.981
sc. media	0.996
congo red	0.997
calcofluor	0.994
grape juice	0.99
sc glycerol media	0.996
exponential growth	0.995
low nitrogen	0.995
low phosphate	0.994
oxidative stress	0.993
heat shock	0.993
hydroxyurea	0.998
sorbitol	0.996
benomyl	0.995
salt	0.991
high calcium	0.999
stationary phase	0.994
