# Supplementary material for: Extensive Transcript Diversity and Novel Upstream Open Reading Frame Regulation in Yeast
Source: G3 (Bethesda). 2013 Feb 1;3(2):343–52. doi: 10.1534/g3.112.003640 (PMC3564994; doi:10.1534/g3.112.003640)
Supplement: RNA-seq data [file supp_3_2_343_v2_index.html]

RNA-seq data 

# Extensive Transcript Diversity and Novel Upstream Open Reading Frame Regulation in Yeast

## RNA-seq data for Waern and Snyder, 2013

http://downloads.yeastgenome.org/published\_datasets/Waern\_2013\_PMID\_23390610/

The SGD directory contains GFF3 files with details of differentialy expressed genes under 17 different growth condtions using RNA-seq and DE-seq analysis in S. cerevisiae, performed by Waern and Snyder (PMID 23390610). See the README document for more information.
